# Intelligent Reflecting Surfaces Enhanced Mobile Edge Computing: Minimizing the Maximum Computational Time

**DOI:** 10.3390/s22228719

**Published:** 2022-11-11

**Authors:** Mubashar Sarfraz, Haya Mesfer Alshahrani, Khaled Tarmissi, Hussain Alshahrani, Mohamed Ahmed Elfaki, Manar Ahmed Hamza, Ali Nauman, Tahir Khurshaid

**Affiliations:** 1 Department of Electrical Engineering, National University of Modern Languages, Islamabad 44000, Pakistan; 2 Department of Information Systems, College of Computer and Information Sciences, Princess Nourah Bint Abdulrahman University, Riyadh 11671, Saudi Arabia; 3 Department of Computer Sciences, College of Computing and Information System, Umm Al-Qura University, Mecca 24382, Saudi Arabia; 4 Department of Computer Science, College of Computing and Information Technology, Shaqra University, Shaqra 11961, Saudi Arabia; 5 Department of Computer and Self Development, Preparatory Year Deanship, Prince Sattam Bin Abdulaziz University, AlKharj 11671, Saudi Arabia; 6 Department of Information and Communication Engineering, Yeungnam University, Gyeongsan 38541, Korea; 7 Department of Electrical Engineering, Yeungnam University, Gyeongsan 38541, Korea

**Keywords:** mobile edge computing, intelligent reflecting surfaces, latency, optimization

## Abstract

Intelligent reflecting surfaces (IRS) and mobile edge computing (MEC) have recently attracted significant attention in academia and industry. Without consuming any external energy, IRS can extend wireless coverage by smartly reconfiguring the phase shift of a signal towards the receiver with the help of passive elements. On the other hand, MEC has the ability to reduce latency by providing extensive computational facilities to users. This paper proposes a new optimization scheme for IRS-enhanced mobile edge computing to minimize the maximum computational time of the end users’ tasks. The optimization problem is formulated to simultaneously optimize the task segmentation and transmission power of users, phase shift design of IRS, and computational resource of mobile edge. The optimization problem is non-convex and coupled on multiple variables which make it very complex. Therefore, we transform it to convex by decoupling it into sub-problems and then obtain an efficient solution. In particular, the closed-form solutions for task segmentation and edge computational resources are achieved through the monotonical relation of time and Karush–Kuhn–Tucker conditions, while the transmission power of users and phase shift design of IRS are computed using the convex optimization technique. The proposed IRS-enhanced optimization scheme is compared with edge computing nave offloading, binary offloading, and edge computing, respectively. Numerical results demonstrate the benefits of the proposed scheme compared to other benchmark schemes.

## 1. Introduction

The future networks will support ultra-reliable and low latency communications and connect billions of devices [1]. These networks will consume very low energy and support high-speed data applications [2]. These networks will merge various technologies and systems such as intelligent reflecting surfaces (IRS) [3], mobile edge computing (MEC) [4], backscatter communications [5], blockchain [6], artificial intelligence [7], non-orthogonal multiple access [8], and a new spectrum [9]. These networks connect ground to air and space for global coverage [10]. However, significant research efforts are required to transition these technologies and systems smoothly to the existing networks. In this regard, researchers are actively investigating the above-mentioned technologies and their performance and integration into the existing networks.

As we enter the era of the “Internet of Things” (IoT), countless devices such as sensor nodes are expected to be linked together [11,12]. However, these devices cannot easily support resource-intensive applications because of the excessive computational latency resulting from their limited computing capabilities. To address this challenge, central computing nodes can be placed near the network’s edge (typically in close proximity to the access points, or APs) [13]. The use of local and edge computing resources for the processing of these computational tasks has the potential to lower the computational time of resource hungry applications, provided that the tasks are off-loaded successfully [14]. MEC is the term used to describe this approach to computing [15,16]. Unfortunately, this MEC paradigm has not yet reached its full potential due to the computation offloading link being imperfect at present. For instance, devices at the cell’s periphery have a notoriously low offloading success rate and/or may experience increased latency when offloading computation rather than performing the work locally. Because of this, these gadgets must rely on their computing power, which is usually insufficient to run resource-intensive programs. That is why it is crucial to boost the MEC systems’ communications performance.

Thanks to recent developments in programmable meta-materials, IRS can be built to increase the spectral as well as the energy efficiency of wireless communications [17,18]. In this context, “IRS” refers to both the controller and the many passive reflecting elements that make up the system [19]. The IRS controller sends instructions to each component of the reflecting system, which then adjusts the amplitude as well as the phase of the reflected signals individually [20,21]. IRS achieves its gain by combining the gains from their virtual arrays and reflection-assisted beamformers. Following that, the reflection beamforming gain is further achieved by adjusting the phase shift induced by each element of the IRS, while the virtual-array gain is obtained by combining both signals, e.g., direct and IRS-reflected signals [22,23]. Bringing together these two types of advantages allows the IRS to maximize the potential of MEC systems, thereby raising the success rate of off-loading devices. As a result of this treatise, we hope to have a better understanding of the IRS and its role in MEC networks.

Extensive work has been carried out on resource optimization and performance analysis in traditional wireless networks. For example, the authors of [24] have proposed multi-objective optimization in NOMA wireless networks to jointly maximize the energy and spectral efficiency of the system. Another paper in ref. [25] has used reinforcement learning to improve the performance of backscatter networks. For short-range information sharing, a multicluster backscatter communication model was developed in particular. This has been followed by a power allocation algorithm that employs the Q-learning technique to minimize network interference. Researchers in ref. [26] have improved the sum rate of small-cell networks through efficient power allocation. They optimized the uplink transmission while ensuring each user’s quality of service requirement and the maximum transmit power constraint at each node. To solve the non-convex optimization problem, a new sequential quadratic programming-based solution has been proposed. The paper in ref. [27] has investigated an optimization problem to maximize the secrecy rate of a NOMA-enabled multi-cell backscatter network by optimizing the reflection coefficient of the backscatter node in the presence of multiple eavesdroppers in each cell, in particular. The optimization problem was formulated as a convex problem and the authors used Karush–Kuhn–Tucker conditions to obtain an optimal solution. Moreover, the researchers in ref. [28] have employed reinforcement learning for backscatter-enabled software-defined heterogeneous networks, and ref. [29] have investigated the energy efficiency of NOMA heterogeneous networks, respectively.

### 1.1. Related Work

At the moment, there are two main categories of MEC systems [4]: those designed for a single user [30,31] and those designed for multiple users [32,33,34]. When it comes to designing MEC systems for a single user, one of the most important performance metrics is referred to as the computation offloading strategy. For a more detailed explanation, see [31], in which a binary offloading strategy is proposed to determine whether a task is going to compute locally by the user equipment (UE) or executed remotely at the MEC server. For data-partitioning-oriented applications, a partial offloading scheme was developed by Wang et al. [30] as a response to this problem; In this setup, some data are being processed at AP referred to as the edge of the network while the rest is processed locally on the mobile device. In addition, interference from other users in practical multi-user systems can degrade the performance of the MEC system by disrupting the radio communications link of the network and the edge server node. Similarly in the scenario of multi-cell, multi-user, for example, to maximize efficiency, Sardellitti et al. [32] proposed a framework for joint optimization of transmit precoding matrices and users’ share of available computing power. On the other hand, in a multi-carrier scenario, Energy-efficient resource allocation across terminals, radio access networks (RANs), and ESs is proposed by Sheng et al. [35]. Moreover, Chen et al. proposed a joint framework for computation offloading and policy for the selection of channels using the principles of classical game theory as their inspiration [36]. Offloading decisions are made at the device level in this type of system. Similarly, in recent years, different algorithms have been proposed for both mobility-aware dynamic scheduling and user association approaches for multi-user, multiple-edge computing nodes [34,34]. Both of these schemes are intended to be implemented shortly. Yang et al. [37] developed a federated learning algorithm that can be executed over-the-air. This was done to further decrease latency, decrease power consumption, and safeguard user privacy in the concept of MEC systems. The extensive computing being offloaded onto devices in challenging communications environments is one that is not fully studied in the literature. So, as per the above literature review, in this paper, we provide evidence that IRS is useful in such circumstances. Let us take a look at how these significant IRS contributions to science were implemented, to continue our previous discussion.

IRS has been extensively studied for its potential uses in wireless communications through channel estimation [38], ergodic capacity analysis [39], and phase shift modeling [40], as well as the phase shift control [41,42,43]. Examples include [41] in which authors proposed a joint framework for phase shift control at IRS and also design a matrix for precoding at the AP, which employs complex semidefinite relaxation and alternating optimization methods to lower transmission power such that the desired signal to interference and noise (SINR) is maintained on the receiver. A more realistic discrete phase shift scenario was added to the studies to better reflect reality. The problem with using the algorithm presented in ref. [41,42] for large-scale IRS is that it requires too much processing power to be practical. Since each group of IRS elements uses the same phase shift coefficient, In order to reduce the amount of extra work required for the IRS channel estimation, Yang et al. [43] proposed a framework for optimal allocation of transmission power and phase shift in OFDM-based wireless systems. After that, in light of the significant scientific contributions described above, we concluded that it was imperative for MEC systems to make use of the advantageous function provided by the IRS.

### 1.2. Motivation and Contribution

This work investigates the IRS-enhanced MEC, designed to improve the system’s computing performance in terms of computational capability and enhance the communication channel capacity. Following that, the computational capability of the devices is enhanced by the concept of a MEC server, whereas the effectiveness of the communication channel is enhanced by introducing the IRS. UEs generally have a finite battery and a limited amount of processing power. UEs are expected to have some data processing capability, even if the processing capability is much slower than the MEC. To achieve this, in this work, for task placement, a concept of partial offloading is considered, which is the process of partitioning a task into two parts and offloading the latter to the MEC server while leaving the former for the UEs to handle locally. Furthermore, the optimal task segmentation was carried out by taking into account the transmission power, phase shift control, and edge computational resources to reduce the overall computational time required by a task. In order to accomplish this, we formulate the joint task computational time minimization problem, which is non-linear and non-convex and is challenging to solve in real-time. To address this, we decoupled the original optimization problem into sub-problems and solved them iteratively until convergence criteria were met. Additionally, the effectiveness of the proposed scheme is demonstrated through numerical comparisons with an exhaustive search and other benchmark schemes.

As mentioned in this paper, we discuss a dynamic approach for task segmentation within the IRS-enhanced MEC network. In particular, the main contribution of this work is as follows:

1. We proposed a task’s computational time minimization problem with joint optimization of computational and communication resources, such as edge computational resource, phase shift control, transmission power, and task segmentation, under energy and system constraints;
2. To address the non-linearity and non-convexity that characterize the original optimization problem, we decoupled the original optimization problem into sub-problems and iteratively solved them. Furthermore, we also drive a closed-form solution for task segmentation and computational resource allocation at MEC;
3. Numerical results are compared to the exhaustive search and other benchmark schemes to demonstrate the efficacy of the proposed scheme. By taking into account, task computational time and energy consumption as performance matrices, numerical results show that the proposed scheme performs epsilon equally to the exhaustive search and outperforms all other schemes.

The rest of the paper is structured as follows. In Section 2, we outline the system model and the problem formulation. In Section 3, we propose the effective framework for optimal allocation of resources. Section 4 contains our simulation results and discussions. The last section of this paper, Section 5, concludes the paper.

## 2. System Model

**Figure 1:**
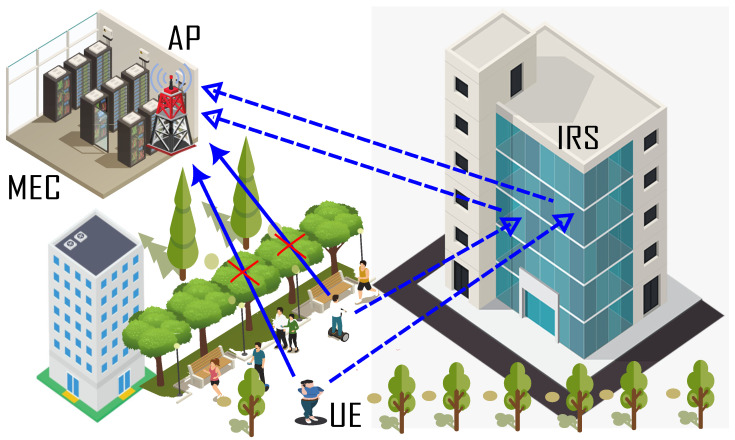
System Model.

In this work, we considered IRS-enhanced mobile edge computing (MEC), where MEC is located at the access point (AP) to provide computational resources to the *N* number of single-antenna users (UE), also known as sensor nodes, which are distributed uniformly over a predefined area in a dense urban environment. AP is equipped with an *M* number of antennas. Sensor nodes typically have limited computational resources and battery life, making them insufficient to handle or compute the massive amounts of data generated by real-time applications. As a result, the system experiences latency. To address this, MEC emerges as a viable solution that offers on-demand extensive computation, allowing these devices to offload their portion of data for extensive computation using binary or partial offloading schemes. In contrast, in a dense urban environment, the communication link between devices and MEC is severely hampered by obstructions such as high-rise buildings. To improve a communication link, we assume an intelligent reflecting surface (IRS) is installed over the building, as shown in Figure 1. The IRS is made up of *K* reflecting elements that intelligently reflect the signal communicated from the user to MEC. Moreover, We use the most adopted model for the task, i.e., $Z_{n}=\left\{s_{n},c_{n}\right\}$, where $s_{n}$ represents the size in terms of bits and $c_{n}$ represents the computational cycle requirement of the task $Z_{n}$. The partial offloading scheme for the assignment of tasks is taken into account in this study. In a scheme that only partially offloads the task, the total task $Z_{n}$ is split in two. Part of the computation is performed locally, while the rest is offloaded directly to the MEC server over a direct connection (UE-MEC) and assisted by IRS (UE-IRS-MEC).

### 2.1. Communication Model

In this section, we introduce the communication model for both direct and assisted links. Let us assume that $g_{n}^{d}\in C^{1\times M}$, $g_{n}^{a,1}\in C^{1\times K}$, and $H\in C^{M\times M}$ as the channel gain coefficients between UE-MEC, UE-IRS and IRS-MEC respectively. Following that $\theta =\mathrm{diag}\{\phi_{1},\phi_{2}...\phi_{K}\}$ diagonal matrix satisfying $∥\phi_{k}∥=1,\forall K$ to ensure fully reflection of the signal from IRS. Furthermore, we assume that UE mobility is not too high, so the channel remains constant for the entire period *T*. Denote that $p_{n}$ is the up-link transmission power of the user. Therefore, data rate $R_{n}$ can be expressed as follows:

(1) $$R_{n}=B\log_{2}\left(1+\frac{p_{n}{\left|y_{n}^{H}\left(g_{n}^{d}+H\theta g_{n}^{a,1}\right)\right|}^{2}}{\sum_{i\neq n}{\left|y_{i}^{H}\left(g_{i}^{d}+H\theta g_{i}^{a,1}\right)\right|}^{2}p_{i}+y_{n}^{H}\sigma^{2}}\right).$$

In Equation (1), $y_{n}$ represents the beamforming vector at the receiving side, and it can be calculated easily using the minimum mean square error. Whereas $\sigma^{2}$ represents the additive white gaussian noise with mean zero and variance 1. Therefore, the time required to offload the portion of the task to the MEC server can be mathematically expressed as:

(2) $$t_{n}^{R}=\frac{\alpha_{n}s_{n}}{R_{n}}.$$

In Equation (2), $\alpha_{n}$ represents the portion of the task offloaded to the MEC server for extensive computation. Furthermore, the energy consumed while offloading the task $Z_{n}$ is expressed as follows:

(3) $$E_{n}^{R}=\frac{p_{n}\alpha_{n}s_{n}}{R_{n}}.$$

### 2.2. Task Computational Model

Specifically, in this work we considered a partial offloading scheme to carry out the computation. Partial offloading splits a task into two parts, with one part being processed locally and the other being sent to a remote MEC for more intensive processing.

#### 2.2.1. Local Computation

Since each UE in a local computation scheme has a finite computational capacity, or $\kappa_{n}^{l}$ (cycles/s), the time spent on the computation can be expressed as:

(4) $$t_{n}^{l}=\frac{\left(1-\alpha_{n}\right)c_{n}}{\kappa_{n}^{l}}.$$

Additionally, the energy consumed while computing the task locally can be expressed as:

(5) $$E_{n}^{L}=\Gamma_{n}\left(1-\alpha_{n}\right)c_{n}.$$

The constant power consumption of a circuit per CPU cycle is denoted by $\Gamma_{n}$, and this value is the same across all devices.

#### 2.2.2. Edge Computing

Access to high-volume computation can be obtained on-demand via MEC, making it a practical solution. Accordingly, users share the server’s computational resources at the MEC. For UE *n*, $\kappa_{n}^{E}$ represents the computing resources allocated to it by MEC. Alternatively, $\kappa^{max}$ represents the MEC server’s absolute computational limit. Additionally, there are two aspects of the total time spent computing the task at MEC: (1) The time it takes for data to be transferred from the UEs to the MEC server, as specified in (2); (2) Processing time on the MEC server. Therefore, the total amount of time the task took to compute on the MEC server can be expressed as:

(6) $$t_{n}^{E}=\frac{\alpha_{n}c_{n}}{\kappa_{n}^{E}}+\frac{\alpha_{n}s_{n}}{R_{n}}.$$

In addition, we presume that the MEC’s battery life is infinite thanks to the constant power from the grid. As a result, the amount of power used by the MEC server while it is processing requests is ignored here. In a similar vein, the authors in ref. [14,16] state that due to the small size of the results, we disregard the transmission time of the results from the MEC server to UE.

### 2.3. Problem Formulation

In this work, we aimed to minimize the computational time of the task by joint optimization of the communication and computational resources among the user and MEC and task segmentation variable. Furthermore, for the ease of simplicity, we define $P=\{p_{1},p_{2},\cdots p_{n}\}$, $\kappa =\{\kappa_{1}^{E},\kappa_{2}^{E},\cdots \kappa_{n}^{E}\}$ and $\alpha =\{\alpha_{1},\alpha_{2},\cdots \alpha_{n}\}$. Following that, the computational time minimization problem can be mathematically expressed as follows:

(7a) $$\begin{array}{cc} P1: & \;\;\min_{\begin{array}{c} P\;,\kappa ,\alpha ,\theta \end{array}}\max\left(\frac{\left(1-\alpha_{n}\right)c_{n}}{\kappa_{n}^{l}},\frac{\alpha_{n}c_{n}}{\kappa_{n}^{E}}+\frac{\alpha_{n}s_{n}}{R_{n}}\right) \end{array}$$

(7b) $$\begin{array}{cc} \; & C_{1}:\frac{p_{n}\alpha_{n}s_{n}}{R_{n}}+\Gamma_{n}\left(1-\alpha_{n}\right)c_{n}\leq E_{n}^{max},\forall n \end{array}$$

(7c) $$\begin{array}{cc} \; & C_{2}:\sum_{n=1}^{N}\kappa_{n}^{E}\leq \kappa^{max} \end{array}$$

(7d) $$\begin{array}{cc} \; & C_{3}:p_{n}\leq P^{max},\alpha_{n}\in \left(0,1\right),\forall n, \end{array}$$

(7e) $$\begin{array}{cc} \; & C_{4}:\left|\phi_{k}\right|=1,\forall k. \end{array}$$

The constraint (7b) guarantees that the total amount of energy used to compute the task will be less than the maximum amount of energy the battery can hold $E_{n}^{max}$, whereas constraint (7c) states the computational resources allocated to UEs at the MEC server should be less than the maximum computational resources. Similarly, constraint (7d) represents the transmission power constraints of the UEs. Following that, phase shift control of IRS elements is ensured by (7e).

In addition, the optimization problem that is discussed in the (7a–7e) is of the mixed-integer, non-convex, and non-linear variety. This is because the rate equation contains a logarithmic function. In order to address this issue, we decoupled the original problem into a series of sub-problems. Moreover, to find the optimal best solution, the sub-optimization problems are then solved iteratively.

## 3. Proposed Solution

In this section, we demonstrate a framework for efficient allocation of computational and communication resources, task segmentation variables and phase shift control of IRS.

### 3.1. Task Segmentation

In the MEC framework, task segmentation is a crucial design parameter that significantly affects overall system performance. Our research takes into account a scheme for partial offloading in which parallel computations are performed both locally and on the MEC server. As a result, there is a monotonic relationship between the local and the edge computation time, as perceived from (4) and (6). The task segmentation variable $\alpha_{n}$ affects both the local and edge computational time. When $\alpha_{n}$ is small, most of the work can be carried out locally at UEs, and the time on each edge is negligible ($t_{n}^{E}\approx 0$). In contrast, when $\alpha_{n}$ approaches its maximum value of 1, most of the work is carried out on the MEC server, resulting in a local computational time that is roughly equal to zeros, i.e., $t_{n}^{L}\approx 0$. A situation exists where the computational edge time is the same as the local computational time or $t_{n}^{L}=t_{n}^{E}$. The following is a mathematical expression of this:

(8) $$\frac{\left(1-\alpha_{n}\right)c_{n}}{\kappa_{n}^{l}}=\frac{\alpha_{n}c_{n}}{\kappa_{n}^{E}}+\frac{\alpha_{n}s_{n}}{R_{n}},$$

(9) $$\frac{c_{n}}{\kappa_{n}^{l}}-\frac{\alpha_{n}c_{n}}{\kappa_{n}^{l}}=\frac{\alpha_{n}c_{n}}{\kappa_{n}^{E}}+\frac{\alpha_{n}s_{n}}{R_{n}}\Rightarrow \alpha_{n}=\frac{\kappa_{n}^{L}A}{c_{n}R_{n}B},$$

whereas $A=R_{n}c_{n}+\kappa_{n}^{E}s_{n}$ and $B=\kappa_{n}^{E}+\kappa_{n}^{L}$. Equation (9) represents the task segmentation expression, which depends on local and edge resources as well as phase shift control. Similarly, the optimal values of these variables result in the optimal values of the task segmentation variable.

### 3.2. Edge Computational Resource Allocation

This section computes the close form solution of edge computation resources assigned to the *n*th UEs task.

**Lemma** **1.** *Under given optimal value task segmentation $\alpha_{n}$, transmission power $p_{n}$ and phase shift of IRS $\theta_{n}$, the computational task minimization problem is convex and mathematically can be expressed as follows:*

(10a) $$\begin{array}{cc} P2: & \min_{\begin{array}{c} \kappa \end{array}}\max\left(\frac{\left(1-\alpha_{n}\right)c_{n}}{\kappa_{n}^{l}},\frac{\alpha_{n}c_{n}}{\kappa_{n}^{E}}+\frac{\alpha_{n}s_{n}}{R_{n}}\right), \end{array}$$

(10b) $$\begin{array}{cc} \; & C_{2}:\sum_{n=1}^{N}\kappa_{n}^{E}\leq \kappa^{max}. \end{array}$$

**Proof.**  Therefore for the ease of simplicity we can define the objective function of (10) as:

(11) $$Q\left(\kappa \right)=\max\left(\frac{\left(1-\alpha_{n}\right)c_{n}}{\kappa_{n}^{l}},\frac{\alpha_{n}c_{n}}{\kappa_{n}^{E}}+\frac{\alpha_{n}s_{n}}{R_{n}}\right),$$

whereas the hessian matrix of (11) can be expressed as:

(12) $$H=\left[\begin{array}{ccc} \frac{\partial^{2}Q}{\partial^{2}\kappa_{1}} & \cdots & \frac{\partial^{2}Q}{\partial \kappa_{1}\partial \kappa_{n}}\\ \vdots & \ddots & \vdots \\ \frac{\partial^{2}Q}{\partial \kappa_{n}\partial \kappa_{1}} & \cdots & \frac{\partial^{2}Q}{\partial^{2}\kappa_{n}}. \end{array}\right]$$

After some mathematical calculation, it was perceived that all the elements of matrix **H** were zeros except for the digital elements, i.e.,

(13) $$\frac{\partial^{2}Q}{\partial \kappa_{n}\partial \kappa_{n^{\prime }}}=\left\{\begin{array}{c} \frac{2c_{n}}{\kappa_{n}^{n^{\prime }}}\;\mathrm{if}\;\;n=n^{\prime }\\ 0=\;\mathrm{otherwise}. \end{array}\right.$$

Equations (13) demonstrate that all of the elements along the diagonal of the matrix **H** have a positive value. Therefore, in accordance with the theorem [44], the objective function mentioned in (11) is convex in nature. As a results, the optimization problem mentioned in (10) is a convex optimization problem. □

**Lemma 2.**  *The optimal allocation of resources at the MEC can be calculated by using Equation (17).*

**Proof.**  According to **Lemma 1**, the Lagrange function of optimization problem (10) can be mathematically represented as:

(14) $$L\left(\kappa_{n}^{E},\lambda \right)=Q\left(\kappa_{n}^{E}\right)+\lambda \left(\sum_{n=1}^{N}\kappa_{n}^{E}-\kappa^{max}\right).$$

Therefore, according to the KKT condition, if ${\kappa_{n}^{E}}^{*}$ and $\lambda^{*}$ is the optimal value of the solution, then we can say that:

(15) $$\Delta Q\left(\kappa_{n}^{E}\right)+\lambda^{*}\left(\Delta \sum_{n=1}^{N}\kappa_{n}^{E^{*}}-\kappa^{max}\right)=0,$$

(16) $$\sum_{n=1}^{N}\kappa_{n}^{E^{*}}-\kappa^{max}=0.$$

Therefore, by solving the above mention equation, the optimal value of computational resource allocation at the MEC can be expressed as follows:

(17) $${\kappa_{n}^{E}}^{*}=\frac{\sqrt{c_{n}}}{\sum_{n=1}^{N}\sqrt{c_{n}}}.$$

□

### 3.3. Transmission Power and Phase Shift Control

Under given the optimal value of task segmentation variable, and computational resources at the MEC server The computational task minimization problem is written in a simplified form as:

(18a) $$\begin{array}{cc} P4: & \min_{\begin{array}{c} P,\theta \end{array}}\max\left(\frac{\left(1-\alpha_{n}\right)c_{n}}{\kappa_{n}^{l}},\frac{\alpha_{n}c_{n}}{\kappa_{n}^{E}}+\frac{\alpha_{n}s_{n}}{R_{n}}\right) \end{array}$$

(18b) $$\begin{array}{cc} \; & C_{1},C_{3},C_{4}. \end{array}$$

The optimization problem that is mentioned in (18) is a non-linear and still non-convex problem due to log function in $R_{n}$. Therefore, to tackle this, we transform the optimization problem into a more easily tackled form by introducing the slack variable $R_{n}^{\prime }$ as follows:

(19a) $$\begin{array}{cc} P3: & \min_{\begin{array}{c} P,\theta ,R^{\prime } \end{array}}\max\left(\frac{\left(1-\alpha_{n}\right)c_{n}}{\kappa_{n}^{l}},\frac{\alpha_{n}c_{n}}{\kappa_{n}^{E}}+\frac{\alpha_{n}s_{n}}{R_{n}^{\prime }}\right) \end{array}$$

(19b) $$\begin{array}{cc} \; & \Phi_{n}\geq {\frac{2^{R_{n}^{\prime }/B}}{\Phi }}_{i}-\Phi_{i},\forall n \end{array}$$

(19c) $$\begin{array}{cc} \; & C_{1},C_{3},C_{4}, \end{array}$$

where $\Phi_{n}=p_{n}{\left|y_{n}^{H}\left(g_{n}^{d}+H\theta g_{n}^{a,1}\right)\right|}^{2}$ and $\Phi_{i}=\sum_{i\neq n}{\left|y_{i}^{H}\left(g_{i}^{d}+H\theta g_{i}^{a,1}\right)\right|}^{2}p_{i}+y_{n}^{H}\sigma^{2}$. Hense the optimization problem mention (19) in convex in nature and thus can be solved by using the convex optimization toolbox, such as CVX, the steps of which are illustrated in Algorithm 1.

**Algorithm 1:** Framework for Optimal Resource Allocation.

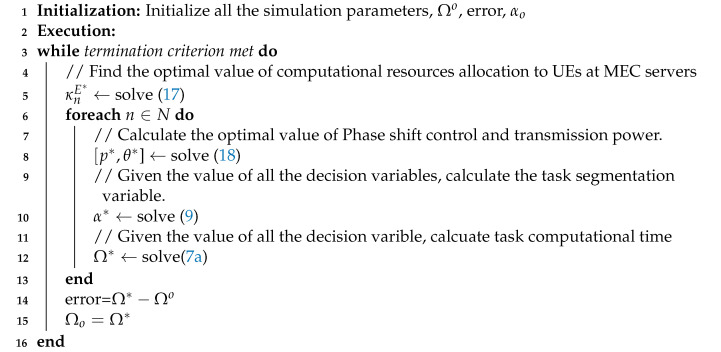



### 3.4. Worst Case Per Iteration Complexity Analysis

The optimization problem mentioned in (19) involves N.K.K.N decision variables and N.N.N.K.K convex constraints. Therefore, according to the authors of [45], the worst case per iteration complexity of can be expressed as $O{\left(N^{2}K^{2}\right)}^{3}\left(N^{3}K^{2}\right)$. Following that, by assuming the $t^{max}$ as the total iterations of the algorithm, the overall complexity of the algorithm can be expressed as $O\left(t^{max}{\left(N^{2}K^{2}\right)}^{3}\left(N^{3}K^{2}\right)\right)$.

## 4. Result and Discussion

Here, we show numerical results to prove the effectiveness of the proposed approach. For this, extensive simulations were executed with the simulation parameters from Table 1. The proposed scheme is also compared to other benchmark schemes, such as binary offloading [46], edge computing, and naive offloading, to ensure it delivers the best performance possible. In the binary offloading method, the entire job is either computed locally or on the MEC server. With edge computing, all UEs offload their processing to the MEC server, while with naive offloading, UEs offload their extensive computation in a random manner.

**Figure 2:**
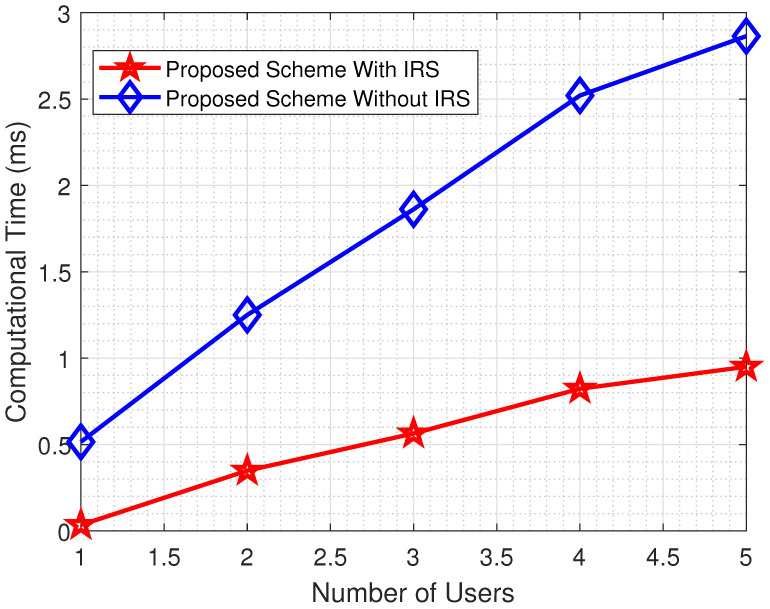
Impact of IRS on system performance.

To begin, it was essential to evaluate the proposed scheme’s performance with and without the involvement of the IRS [12]. After that, we compared it with an exhaustive search as well as various other benchmark schemes. In order to accomplish this, Figure 2 shows the IRS’s effects on the performance of the system. In a dense urban environment, the communication link between the UEs and MEC servers is highly tempered due to blockage, e.g., high-rise buildings. Therefore, as a result of poor channel conditions, achievable data decrease, which has a significant impact on the amount of time required to complete the task. Thanks to IRS, that emerges as a practical solution to assist the existing terrestrial communication system. In order to improve the channel gain, the IRS elements perform an intelligent signal reflection from the UEs in the direction of the MEC servers. As a consequence of this, the achievable data rate increases, and the amount of time required to offload the task decreases, as shown in Figure 2.

**Figure 3:**
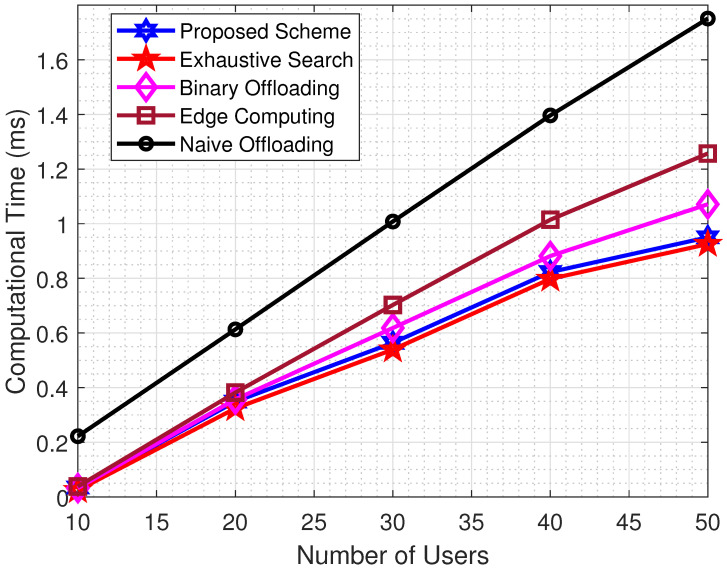
Computational Time vs Number of Users.

The results in Figure 3 demonstrate the comparative analysis of the proposed scheme with exhaustive search and other benchmark schemes by considering computational time as a performance metric. Results show that the proposed scheme produces epsilon equal results as compared to exhaustive search and outperforms all others. Outcomes reveal that, for a small number of users, the performance of the proposed schemes follows one another. In contrast, as the number of users increases in the system, the proposed scheme performs better than others. This trend is because edge computational resources are shared among the number of connected users. For a large number of users, the portion of resources allocated to each user is minute as compared to a small number of users as a result of latency in introducing a system. In contrast, in the proposed scheme, the task is optimally divided into two parts by taking into account the edge computational resources and channel characteristics. One portion of the task is computed locally, while the other is offloaded to MEC for extensive computation.

**Figure 4:**
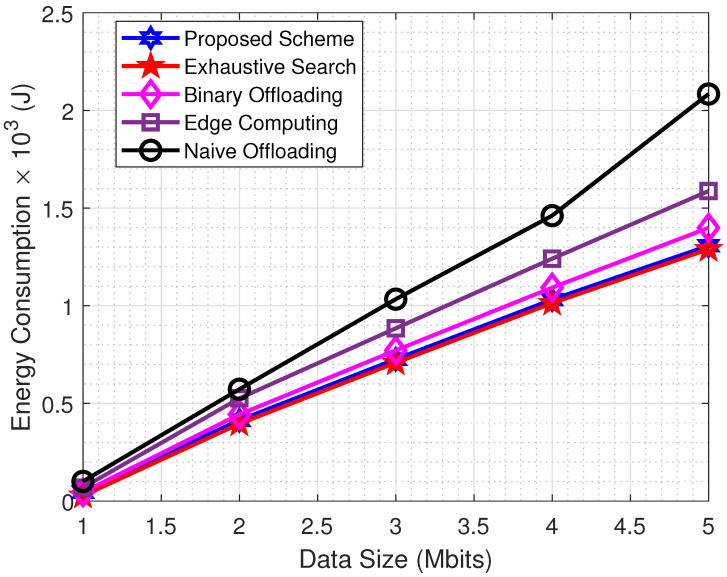
Energy Consumption vs. Data Size.

The amount of data being processed is an essential component of the edge computing paradigm and has a considerable impact on the overall performance of the system. In order to demonstrate the usefulness of the proposed scheme, several simulations were carried out using sets of varying data sizes. The effectiveness of the proposed scheme is demonstrated by the results in Figure 4. In addition, the results demonstrate that the time required for edge computation increases at an exponential rate when the data size increases. This is due to the fact that the total computational time in edge computing is primarily determined by the achievable data rate, denoted by $R_{n}$, as well as the number of bits, as specified in (2). In the context of edge computing, the value of the task segmentation variable is $\alpha_{n}=1$. That is to say, all of the bits are offloaded to MEC severe. In that case, an edge computing scheme is not an acceptable method for task computation because of a direct relationship between the amount of time it takes to offload data and the number of bits involved. When compared to other methods, optimal task segmentation always performs better and emerges as a viable solution for problems involving large amounts of data.

**Figure 5:**
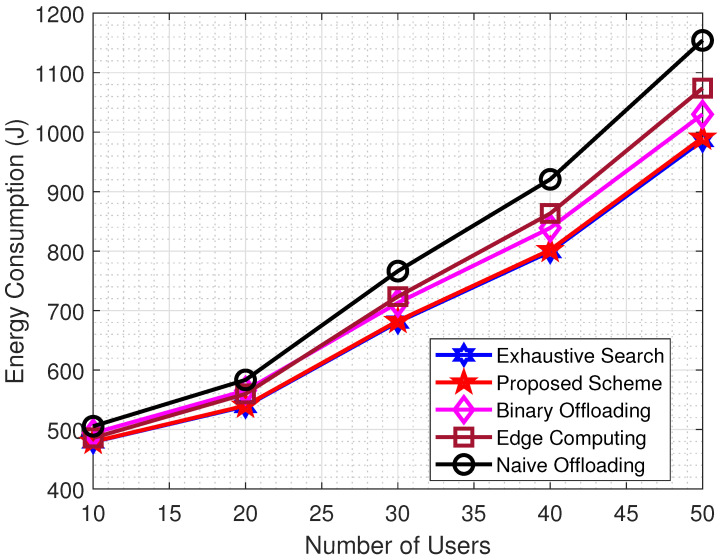
Energy Consumption vs. Number of Users.

Sensors are an essential component of any intelligent control system that collect the necessary data from applications that operate in real-time. The most recent improvement to the 5G/6G communication system has allowed for the deployment of additional sensor nodes. These sensor nodes, in addition to having a limited capacity for computation, also have a finite battery life, which is a factor that bottlenecks the performance of the system. The simulation was carried out so that the efficacy of the proposed scheme can be evaluated with regard to the amount of energy that it would require to compute the task. The effectiveness of the proposed scheme is illustrated by the numerical results in Figure 5. The results show that, for a small number of users, the proposed scheme follows the others. However, as the number of users increases, the proposed scheme begins to perform better due to its optimal task segmentation into two portions. In the proposed scheme, only a small portion of the task is computed locally, resulting in lower energy consumption when compared to binary or edge computation schemes, in which the entire task is offloaded to MEC for extensive computation, resulting in higher energy consumption.

## 5. Conclusions

IRS and MEC are the two promising technologies for low latency and high energy efficiency communications in future networks. This paper has provided a new framework for minimizing the maximum computational time of user tasks using an IRS-enhanced MEC network. In particular, this framework has simultaneously optimized the task segmentation and transmission power of users, phase shift design of IRS, and computational resource of edge, respectively. Since the problem was non-convex and coupled with multiple variables, it was hard to obtain the joint solution directly. Therefore, it was decoupled into sub-problems first and then solved through convex optimization methods. The proposed scheme was validated and compared through numerical results. Results show that the proposed IRS-enhanced MEC network performs significantly better than the other benchmark schemes in terms of computational time and energy consumption. This work can be extended in several ways. For example, we can use aerial IRS such that the IRS can be equipped with a drone. Moreover, we can integrate non-orthogonal multiple access to the same system model, where the existing model can act as a benchmark.

## Figures and Tables

**Table 1:** Simulation Parameters.

Name	Symbol	Value
Transmission Bandwidth	*B*	20 MHz
Maximum Transmission Power	$P^{max}$	0.1 W
Noise Power	$N_{o}$	−173 dBm
Static Circuit Power	Γ	90 W/Gcycle
Maximum battery Capacity	$E^{max}$	$10^{3}$ J

## Data Availability

Not applicable.
